# Time to Consider Potassium Intake in Saudi: A Cross-Sectional Assessment Using 24 h Urinary Excretion

**DOI:** 10.3390/nu17203227

**Published:** 2025-10-14

**Authors:** Salwa Ali Abdullah Albar, Merfat Abdulrahman Almaghrabi

**Affiliations:** Food and Nutrition Department, King Abdulaziz University, P.O. Box 80200, Jeddah 21589, Saudi Arabia

**Keywords:** dietary intake, fruit, vegetables, dietary potassium, sodium, potassium ratio, urinary excretion, Saudi Arabia

## Abstract

**Background:** Evaluating potassium intake can be a powerful tool in epidemiologic studies to reduce the burden of noncommunicable diseases (NCDs). In Saudi Arabia, NCDs are responsible for 35% of deaths in 2023. Monitoring people’s potassium intake can be a powerful tool to reduce the burden of NCDs. There is a significant lack of information on potassium intake. The aim is to assess potassium intake using 24 h urinary excretion; to investigate the urinary sodium-to-potassium (Na/K) excretion ratio among Saudi adults; and to explore other lifestyle factors that influence potassium intake. **Methods:** A cross-sectional survey was conducted among young adults (19–29 years old) residing in Jeddah, Saudi Arabia. Data collection included a self-reported questionnaire regarding participants’ general attitudes and practices related to potassium consumption (*n* = 600) of whom 173 participated in 24 h urine collection. Descriptive analyses and regression models were used to evaluate the associations between urinary potassium excretion (mmol/24 h), daily potassium intake (g/day), and the Na/K ratio (dependent variables), and descriptive variables such as age and gender (predictor variables). A *p* value < 0.05 indicated statistical significance for all tests. **Results:** The mean urinary potassium excretion was 48.6 ± 23 mmol/24 h, equivalent to a mean daily potassium intake of 1.9 ± 0.89 g/day, and only 4.1% of participants met the World Health Organization-recommended potassium intake of ≥90 mmol/day (≥3.90 g/day). The average potassium intake was significantly lower in females compared with males by 0.52 g (95% CI: −0.78 to −0.25; *p* < 0.001). Physical activity was also a significant factor, associated with both urinary potassium excretion (*p* = 0.039) and intake (*p* = 0.006). Besides the low potassium intake, the mean Na/K ratio was 3.2 ± 1.4, and the ratio differed significantly by physical activity habits (*p* = 0.050). Only 13% of participants consumed fruit 5–7 days per week (mean portion intake 1.4/day; 95% CI: 1.3–1.5), and 34.7% consumed vegetables 3–4 days per week (mean portion intake 1.5/day; 95% CI: 1.3–1.5). These findings reflect low adherence to recommended fruit and vegetable intake in the study population. **Conclusions:** The findings of this study can be used to create evidence-based nutritional strategies to help people achieve the recommended potassium intake. The study underscores the need for more research on potassium intake across Saudi Arabia.

## 1. Introduction

Worldwide, noncommunicable diseases (NCDs) are the main contributors to mortality and morbidity [1]. NCDs are the leading cause of death in Saudi Arabia; according to a 2023 report, around 22,000 Saudis die every year from NCDs, which account for 35% of deaths, many of which occur between 30 and 70 years of age [2]. In 2024, the prevalence of leading NCDs in Saudi Arabia is higher than previously reported in national estimates [3]. Heart disease, stroke, kidney disease, and diabetes mellitus are the main contributors to the NCD burden. However, high blood pressure (hypertension) is a major risk factor for cardiovascular diseases, especially coronary heart disease, stroke, and renal failure. The prevalence of hypertension is 34% among Saudi adults, in addition to a high prevalence of obesity and diabetes (40.6% and 39.5%, respectively) [4,5]. As in many parts of the world, NCDs cost Saudi Arabia around 91.6 billion Saudi Riyal (USD24.4 billion) every year to provide healthcare, social care, and welfare support [2]. According to the World Health Organization (WHO), 80% of premature heart disease and diabetes, as well as 40% of cancers, can be prevented by eliminating their risk factors [1,4].

Evaluating potassium intake can be a powerful tool in epidemiologic studies to reduce the burden of NCDs [1,6,7]. Potassium (K+) is an essential nutrient naturally present in many foods and obtained primarily from fruits and vegetables; it helps maintain intracellular fluid volume and electrolyte balance, regulates muscle and nerve function, and supports cardiovascular health [1,6]. Epidemiologic and clinical studies have shown that adequate potassium intake is associated with lower risk factors for several chronic diseases—including reduced blood pressure and lower risk of stroke and type 2 diabetes—and with reduced cardiovascular disease (CVD) mortality [6,8,9,10,11]. Thus, potassium intake has been inversely related to NCD risk. Historically, potassium intake was very high—exceeding 200 mmol/day—in preagricultural (hunter gatherer) and early agricultural diets [12]. However, modern diets—often high in processed foods and low in fresh fruits and vegetables—have led to a significant decrease in potassium intake across many populations, including Saudi Arabia [7,12]. This decline is largely attributed to urbanization and lifestyle transitions, which have altered dietary patterns and been accompanied by increasingly sedentary behaviors [13].

A systematic review and Bayesian meta-analysis of 104 studies from 52 countries reported a global mean potassium intake of 2.25 g/day (57 mmol/day), with the highest intakes in Eastern and Western Europe and the lowest in East Asia (mean intake 1.89 g/day); only 31% of the global population had an estimated potassium intake of >2.5 g/day [14].

Urinary potassium excretion reflects broader lifestyle patterns, including dietary intake, physical activity, and demographic factors. Among these, dietary intake is considered the primary determinant, as approximately 85% of ingested potassium is excreted in urine. Higher consumption of potassium-rich foods such as fruits, vegetables, fish, milk, nuts, and legumes, and lower consumption of low-potassium diets dominated by ultra-processed foods, are strongly associated with greater urinary potassium excretion [15]. Physical activity also influences potassium excretion through sweat losses, altered renal handling, and hormonal regulation [16,17]. In addition, other lifestyle factors, including socioeconomic status, smoking, and alcohol consumption, have been linked to variations in potassium intake and excretion [17,18,19].

A recent report by the WHO and the Food and Agriculture Organization found that a majority of countries fall short of the recommended daily potassium intake of 70–80 mmol/day [20]. Even fewer countries, such as the United Kingdom, Spain, Mexico, and Belgium, report average potassium consumption of 90 mmol/day. Data from the National Health and Nutrition Examination Survey indicate that the average daily potassium intake was 3016 mg for men and 2320 mg for women in the United States [21,22]. Lower potassium intake has been associated with a number of NCDs and risk factors, such as blood pressure, hypertension, and stroke, whereas higher levels of consumption may be protective against these diseases [1,23].

WHO guidelines and recent studies have highlighted the importance of evaluating the sodium-to-potassium (Na/K) intake ratio, as this index is considered more reliable than assessing either sodium or potassium intake alone. A Na/K ratio ≤ 1.0 is reported to be optimal for preserving cardiovascular health [24,25,26]. This ratio results from consuming at least 3510 mg/day of potassium and no more than 2000 mg/day of sodium [27]. Thus, understanding the connections between nutrients and their relationships to one another within the human body is important for preventing such diseases.

Objective estimates of potassium intake from 24 h urine collections are considered accurate and reliable compared with subjective estimates based on 24 h dietary recalls, which may lead to over- or underestimation of actual intake [28,29]. There is limited evidence using 24 h urine collections to assess potassium intake in Saudi Arabia. To our knowledge, there are only two studies that evaluated potassium intake among the Saudi population: one conducted among 25 young soccer players in Riyadh [30] and another among adults in northern Saudi Arabia [31]. Therefore, this study aims to assess 24 h urinary potassium excretion and other lifestyle factors that influence potassium intake, and to investigate the urinary Na/K excretion ratio among Saudi adults.

## 2. Materials and Methods

### 2.1. Study Design and Participants

This cross-sectional study was conducted among residents of Jeddah, Saudi Arabia, between October and December 2024. The sample size was calculated using Cochrane’s formula: *n* = z^2^ × *p* × (1 − *p*)/e^2^, with z = 95% confidence level (z = 1.96); *p* = estimated proportion of the population that presents the characteristic *p* = 0.5; and e = margin of error (e = 0.05 or 5%). The required sample size was 384 participants; adding a 25% attrition rate, a total of 480 participants were required for estimating a proportion of the general dietary behaviors questionnaire.

Based on the WHO recommendation, a minimum of 120 individuals per specific age and gender group is required (this number is based on the expected mean and standard deviation of potassium excretion from the previous literature which is necessary for quantitative analysis). To compensate for the anticipated high rate of participant dropout and incomplete urine sample collection, which are common challenges in this type of study [32], a total of 240 participants were invited from the initial sample pool to participate in the 24 h urine collection.

A convenience sample of 600 young adults (19–29 years old) was recruited via email and social applications such as WhatsApp and Telegram to assess participants’ eligibility, general characteristics, and practices related to general dietary behavior that related to potassium intake. Internet penetration in Saudi Arabia reached 99% of the population in 2024, reflecting an exponential increase. Simultaneously, social media use is nearly universal, with 94.3% of the population identified as active users [33]. Participants who fit the study inclusion criteria were then asked to take part in the urine execration collection, and we stopped inviting them after reaching 240 participants (this was achieved after inviting 500 participants). Thus, the response rate for the urine execration portion was 48%.

The exclusion criteria were females who were pregnant or lactating and participants who reported chronic diseases or long-term prescription medication use. Moreover, participants undergoing treatment with diuretics were also excluded because loop diuretics alter potassium excretion by inhibiting reabsorption in the thick ascending limb, which can lead to an inaccurate assessment of participants’ true potassium status [34]. The study details were explained so that participants understood why the research was being conducted and what it would involve. The participants were informed that participation was voluntary and that they could withdraw at any time without giving a reason.

A total of 240 participants agreed to provide a 24 h urine sample; however, 24 did not return their samples. An additional 38 participants were excluded because of improper collection procedures, such as failing to complete a time sheet, omitting the first-morning specimen, or providing a urine volume of less than 500 mL. Five further samples were also excluded based on low urinary creatinine levels: three women with creatinine excretion less than 4 mmol/day and two men with creatinine excretion less than 6 mmol/day. Thus, urinary excretion data from 173 participants were included.

The study was conducted in accordance with the Declaration of Helsinki, and the Ethical Committee of King Abdulaziz University, Unit of Biomedical Ethics, approved the protocol (Ethics reference: 167–22). Written informed consent was obtained from all participants.

### 2.2. Data Collection and Study Procedures

An electronic semi-structured questionnaire was developed by adapting items from validated international surveillance instruments, including the WHO STEPS diet module [35,36]. It was translated into Arabic using forward–back translation procedures. Content and cultural validation were performed by a panel of experts. Following this review, a pilot study was conducted with 27 participants to refine the instrument prior to its use with the main sample; based on the pilot, some items were refined, and the pilot data were excluded from the final analysis. The questionnaire demonstrated acceptable internal consistency, as indicated by Cronbach’s alpha coefficient of 0.70. The final instrument comprising information about sociodemographic characteristics (age, gender, education, and income) and health characteristics (physical activity, smoking, and eating habits) was collected via a self-reported survey. The participants were asked about the frequency of consuming fruits, vegetables, fast food, ultra-processed foods, and snacks as meals. All items were multiple-choice questions.

The participants who agreed to provide a 24 h urine sample were asked to attend an interview, which was conducted by trained researchers in the university research center. Systolic and diastolic blood pressure (BP) measurements using a random-zero sphygmomanometer were taken four times for each participant (twice at each of two visits), and the mean was used for analysis. Measurements were taken after the participant had rested in a seated position for 15 min, with 3 min of rest between measurements.

### 2.3. Collection of 24 h Urine

For 24 h urine data collection, this study followed the WHO protocol for estimating potassium via urinary excretion [32]. Trained researchers provided participants with verbal and written instructions and sterile urine-collection bottles and answered any questions. The participants were instructed to start the 24 h urine collection from the second morning specimen and continue until the first specimen of the following day. The female participants were instructed to collect urine on non-menstruating days.

The participants completed a time sheet, recording the date of the urine sample and the start and end times, and reported any urine volume missed during the collection period. A 24 h urine sample was considered complete if the total urine volume was >500 mL, the collection period was >20 h, and (for female participants) no menstruation occurred during the collection. Analysis of the 24 h urine samples was conducted at certified laboratories.

The urinary potassium samples were transported in portable thermoelectric coolers and analyzed in certified laboratories using the ion-selective electrode (ISE) method with an Abbott ARCHITECT c8000 Chemistry Analyzer (Abbott Diagnostics, Abbott Park, IL, USA), following the manufacturer’s protocols. This method is a standard and validated laboratory technique for electrolytes, specifically used to quantify urinary potassium (K) [37].

### 2.4. Statistical Analysis

Descriptive statistics were used to summarize participants’ general characteristics. For continuous variables (e.g., age and weight), means and standard deviations (SDs) were used, and the Shapiro–Wilk test was used to assess normality. For categorical variables (e.g., education level and smoking status), the number (*n*) and percentage (%) in each category were reported. To test the proportion of differences between males and females in categorical variables (education, smoking, and body mass index [BMI] classification), Pearson’s chi-square test was used. For continuous variables, independent two-sample t-tests were used to compare genders.

BMI was calculated as weight (kg) divided by height (m^2^). The participants were then classified according to WHO adult guidelines as underweight (BMI < 18.5 kg/m^2^), normal weight (18.5 to <25 kg/m^2^), overweight (25 to <30 kg/m^2^), and obese (BMI ≥ 30 kg/m^2^). Guidelines from the American Heart Association were used to categorize blood pressure (BP): normal BP defined as systolic BP <120 mm Hg and diastolic BP <80 mm Hg; elevated as systolic 120–129 mm Hg and diastolic <80 mm Hg; and high BP when systolic ≥130 mm Hg or diastolic >80 mm Hg [38].

To convert urinary output to dietary potassium intake, urinary K excretion (mmol/day) was multiplied by 39/1000 g (1 mmol K = 39 mg), based on WHO conversion [38]. Associations between urinary K excretion (mmol/24 h), daily potassium intake (g/day), and the Na/K ratio (dependent variables) and descriptive predictors such as age and gender were investigated using regression (model 1).

In addition, associations of these dependent variables with BMI, education, income, physical activity, BP, and fruit and vegetable consumption were investigated using regression (model 2), adjusted for age and gender. One-sample t-tests were performed to test whether the mean potassium intake for the total sample, and after stratification by gender, differed from the WHO recommendation of ≥3.51 g/day. Statistical analyses were carried out using Stata Statistical Software, Release 12 (StataCorp, College Station, TX, USA) [39]. A *p* value < 0.05 indicated statistical significance for all tests.

## 3. Results

### 3.1. Sample Characteristics

Table 1 presents the study participants’ general characteristics overall and stratified by gender. 

A total of 600 adults participated in the study; 60.1% were female. The mean age for the total sample was 23 ± 3 years. Significant differences were found between males and females in mean age (Coef. = −0.74; 95% CI: −1.2 to −0.2; and *p* = 0.004). The majority of participants were of normal weight (62%), and there were significant differences in the proportion of males and females across BMI categories (*p* < 0.001). Most of the sample was single (85%), and 61.5% held bachelor’s degrees. A total of 30.7% reported a family income ranging from 10,999 to 20,000 SAR, followed by 26% with an income range from 5000 to 10,000 SAR. Furthermore, significant differences were observed between males and females in social status, education level, and family economic status.

Most of the sample (84.7%) had no diagnosed health issues, and 25% were smokers. There were significant differences between genders in smoking status and health issues. Significant differences between males and females were also found in physical activity (*p* < 0.001). However, no significant differences were found between genders in BP readings or in following a special diet. Regarding the general characteristics of the participants who provided 24 h urine samples, the study included 173 individuals, 58.4% of whom were males. Their average age was 23.8 ± 3.3 years, with a significant difference observed between males and females in their mean age (mean difference 1.2 years; *p* = 0.018).

There were no significant differences found between males and females in education level, economic status, or blood pressure. However, a significant difference was noted in the proportion of males to females regarding physical activity levels (*p* = 0.003).

### 3.2. General Dietary Behavior

Table 2 illustrates participants’ general dietary behavior. 

Only 13% of participants consumed fruits 5–7 days per week, and nearly half of the sample (48.9%) consumed fruits twice per week, with an average portion size of 1.4 (95% CI: 1.3, 1.5) portions. While 34.7% of participants consumed vegetables 3–4 days per week, 29% consumed vegetables twice per week. The average portion of vegetable consumption was 1.5 (95% CI: 1.3, 1.5) portions. There was a significant difference between males and females in the frequency of fruit and vegetable consumption (*p* < 0.001 and *p* = 0.005, respectively). The frequency of fast-food consumption among participants showed that 34.8% consumed fast food “rarely,” followed by 26% who consumed it “once a week.” Processed food consumption data indicated that 36% of participants consumed it “often,” followed by 33% who consumed it “sometimes.” Significant differences were found between genders in the frequency of fast-food and processed food consumption (*p* = 0.008 and *p* = 0.014, respectively). The general dietary behavior observed in the subsample (*n* = 173) followed a similar trend to that of the main sample.

### 3.3. Twenty-Four-Hour Urinary Volume, Potassium, and Equivalent Dietary Potassium (Supsample)

The average urinary volume excretion was 1642.8 mL/day, and there were significant differences between males and females in urinary volume excretion (mL/24 h), with females having significantly lower urine volume than males (coef. −13.3; 95% CI: −20.1, −6.5; and *p* = 0.014). The mean potassium excretion was 48.6 ± 23 mmol/24 h, which is equivalent to a mean daily potassium intake of 1.9 ± 0.89 g (median: 2.02 g; IQR: 1.2, 2.2). Potassium intake in females was significantly lower than in males by −0.52 g (95% CI: −0.78, −0.25; *p* < 0.001). Only 4.1% of the study participants met the recommended potassium intake levels of >90 mmol/day (>3.90 g/day), as advised by the WHO.

Furthermore, physical activity habits were significantly associated with urinary potassium excretion (mmol/24 h) and daily potassium intake (g) (*p* = 0.039 and *p* = 0.006, respectively), after adjustment for important confounders (age and gender). Participants who consumed more than five portions of fruits and vegetables had significantly higher urinary potassium excretion by 12.9 mmol/24 h (95% CI: 6.6, 19.2; *p* < 0.001) and significantly higher daily potassium intake by 0.50 g (95% CI: 0.26, 0.75; *p* < 0.001).

The average sodium-to-potassium (Na/K) ratio was high at 3.2 ± 1.4, (median: 2.95; IQR: 2.2, 3.9), and the ratio was significantly different according to physical activity habits (*p* = 0.050). However, no significant associations were observed between age, BMI, BMI category, education level, income status, or any other dietary behavior and average urinary potassium excretion (mmol/24 h), daily potassium intake (g), or the Na/K ratio (Table 3).

## 4. Discussion

Assessing current dietary intake is fundamental to crafting evidence-based nutritional strategies that enable individuals to meet recommended nutrient intakes. To our knowledge, this is the first study conducted in the Western Province and one of only two across the Kingdom to assess potassium status using the gold-standard 24 h urinary excretion method. The data reveals a critical nutritional shortfall in the population. Only 4.1% of participants met the WHO-recommended potassium intake level (≥90 mmol/day) ≥3.51 g/day, with an average potassium intake of 1.9 ± 0.89 g (median intake of 2.02 g (IQR: 1.2, 2.2)), a prevalence that is substantially low. The Na/K ratio was high at 3.2 ± 1.4, which strongly indicates a high risk for hypertension and cardiovascular disease within this young population. Furthermore, findings reveal a significant and positive association between physical activity and fruit and vegetable consumption with both urinary potassium excretion and estimated daily potassium intake. This precisely quantifies the linkage between these healthy lifestyle components and overall potassium status.

In Saudi Arabia, only two studies to date have used 24 h urinary potassium excretion as a tool to assess potassium intake, which were conducted in the Riyadh and Al-Jouf regions. Findings from the Riyadh region reported an optimal urinary potassium concentration of 64.3 ± 23.7 mmol/L [30], likely due to the specific nature of the study population, as participants were professional soccer players who were more likely to adhere to optimal dietary intake. Additionally, the study included only 25 players, a small sample size that limits generalizability. On the other hand, in the Al-Jouf region (*n* = 392; aged 25–64 years), the average potassium intake was 2.3 g/day 4 [31].

Similar to our findings, a similar trend was observed in the Gulf region. A nationally representative survey of 569 Omani participants found that the average population potassium intake was 2.36 ± 1.46 g/day, with fewer than 10% meeting the adequate intake level. Significant gender differences were also observed, with females having a lower potassium intake (2.27 ± 1.46 g/day) than males (2.53 ± 1.45 g/day) [15]. A similar finding was reported in the UAE (*n* = 190; aged 20–60 years), where the mean urinary potassium intake was 2.5 ± 0.615 g/day, with significant differences by gender (*p* = 0.007) [40]. This difference may be attributable to the greater energy intake among males [15] as well as the higher physical activity observed in this study.

Most of the world’s population consumes less than the recommended level of potassium [1]. A systematic review and meta-analysis that included 104 studies from 52 countries examined potassium intake assessed by 24 h urinary excretion, dietary methods, or spot urine samples. The mean global potassium intake was found to be 2.25 g/day (57 mmol/day) [14]. These global data indicate significant regional variation, with the highest mean intakes observed in Eastern and Western Europe at 3.53 g/day (95% CI: 3.05–4.01 g/day) and 3.29 g/day (95% CI: 3.13–3.47 g/day), respectively, while East Asia reported the lowest mean intake at 1.89 g/day (95% CI: 1.55–2.25 g/day). Globally, only 14% (95% CI: 11–17%) of the population reached the higher threshold of 3.5 g/day [14].

The general dietary behavior of this study’s participants shows that only 13% consumed fruits 5–7 days per week, with an average portion intake of 1.4 portions/day (95% CI: 1.3, 1.5), and 34.7% consumed vegetables 3–4 days per week, with an average portion intake of 1.5 portions/day (95% CI: 1.3, 1.5). This is significantly lower than the WHO recommendation of at least 400 g (five portions) of fruits and vegetables per day [23] and the USDA recommendation of approximately 4.5 cups/day (2 cups of fruit + 2.5 cups of vegetables) for an average 2000 kcal/day diet. At the same time, a considerable proportion of participants—35%—consumed fast food twice per week, and 36% consumed processed food more often. Changes in lifestyle and dietary patterns have led to a notable rise in the intake of various sodium-rich foods [41,42]. This is consistent with current dietary trends in Western populations, where there is a steady decrease in fruit and vegetable intake and an increase in the consumption of processed (sodium-rich) foods [22]. This results in reduced potassium intake and increased sodium intake; the higher the sodium intake, the greater the urinary potassium loss. Thus, the ratio of sodium to potassium intake may be more important than either nutrient alone [7].

Although potassium intake has received less attention than sodium intake as a public health strategy to reduce the risk of CVDs, results from the SSaSS trial showed that increasing potassium intake may be of greater importance for public health strategies. Increasing potassium by 57% (803 mg) and reducing sodium by 8.1% (350 mg), without modifying participants’ dietary quality, resulted in a 3.34 mmHg reduction in systolic blood pressure [43,44]. A cluster-randomized controlled trial of potassium-enriched salt conducted in Taiwan reported a significant reduction in cardiovascular mortality in the experimental group. Therefore, the Na/K ratio has been reported to be a superior metric compared to either sodium or potassium alone in relation to blood pressure prevention [20,25,45,46,47].

In the current study, the Na/K intake ratio was as high as 3.16 ± 1.41, which is consistent with findings from epidemiological and clinical studies across various regions and populations worldwide. In an Omani study, the average urinary Na/K ratio was 3.3, with no difference by gender [15]. China reported a high average Na/K ratio of 3.93 (95% CI: 3.31–4.64), while the Netherlands and the United Kingdom reported lower levels at 1.60 (95% CI: 1.27–1.90) and 1.60 (95% CI: 1.33–1.87), respectively. Globally, the average Na/K ratio was 2.88 (95% CI: 2.56–3.23), with significant regional differences: the highest ratios were reported in East Asia (3.85; 95% CI: 3.04–4.81) [48], Eastern Sub-Saharan Africa (3.42; 95% CI: 2.12–5.52), and South Asia (3.39; 95% CI: 2.54–4.45) [14]. In contrast, the lowest Na/K ratios were found in Western Europe (1.68; 95% CI: 1.46–1.92) and Eastern Europe (1.82; 95% CI: 1.23–2.48) [14]. There are no accepted guidelines for the optimal Na/K ratio; however, the WHO suggests that meeting individual intake guidelines for both sodium and potassium would result in a ratio of approximately 1.0 [1]. Another study has suggested that a ratio less than two may be an appropriate goal for reducing blood pressure and CVD risk [49,50].

The observed high Na/K ratio in our study population is highly concerning from a public health perspective. Evidence demonstrates that a high Na/K ratio is a better predictor of elevated blood pressure and subsequent CVD mortality than sodium intake alone. This indicates that the population is facing a profound dual dietary imbalance (excessive sodium intake coupled with insufficient potassium intake). This combined imbalance places the individuals in this study at a substantially increased, quantifiable risk for developing hypertension and other CVD complications, necessitating urgent targeted nutritional interventions to reduce CVD burden in Saudi Arabia.

Similar to the findings of this study, engagement in physical activity significantly influences urinary potassium excretion by altering electrolyte balance, leading to reduced urinary sodium and increased urinary potassium through hormonal regulation [30]. One study shows that there are high potassium losses during heavy physical activities in hot climates [51]. Although socioeconomic status did not influence potassium excretion in this study, many studies found that lower urinary potassium excretion was reported among those with the greatest socioeconomic disadvantage compared with higher socioeconomic groups [19]. Another study found that urinary potassium excretion was lower among individuals with lower income and education levels [52]. Evidence from Italy also indicated that individuals in low-skilled employment had lower potassium excretion than those in senior managerial roles [53]. In addition, lower household expenditure has been associated with reduced potassium intake and higher Na/K ratios. Taken together, these studies suggest that socioeconomic status may play an important role in shaping dietary patterns, potassium intake, and overall diet quality [18,54].

Our study has several strengths. It is the first study conducted in the Western Province of Saudi Arabia and the second conducted among the general population that included both genders. It used the gold standard for measuring urinary excretion, applying the WHO’s current preferred technique for estimating potassium consumption [32]. Furthermore, this study collected information on participants’ general lifestyle and dietary behaviors related to potassium intake. On the other hand, some limitations exist in this study. First, the cross-sectional nature of the study prevented the determination of direct association. The study was conducted on a relatively small sample size and covered a narrow age range (19–29 years) in Jeddah, which restricts the generalizability of the results to the nationwide scale. In addition, the use of a convenience sampling technique meant that urine samples were collected from volunteers, which may have introduced self-selection bias. Although we administered a self-reported questionnaire on participants’ general attitudes and practices to explore lifestyle factors influencing potassium intake, the dietary behavior data were reported only in general terms and therefore do not provide a reliable estimate of actual potassium intake. Finally, relying on a single 24 h urine collection may not adequately reflect typical potassium excretion, since multiple samples are generally needed to account for daily variations in intake. However, applying such an approach would have significantly increased the participants’ burden and workload.

## 5. Conclusions

This study assessed 24 h urinary potassium excretion among young Saudi adults aged 19–29 years, addressing a critical gap in regional nutrition research. The findings establish baseline values for this age group and reveal insufficient potassium intake and low adherence to international recommendations, accompanied by elevated sodium-to-potassium ratios. This imbalance may have significant and immediate implications for the prevalence of CVDs and other non-communicable diseases within this young population.

The study provides direct evidence that can aid the health professional in crafting targeted, evidence-based nutritional strategies to mitigate this cardiovascular risk. It is imperative that future public health programs focus on increasing fruit and vegetable consumption to boost population potassium intake and prioritizing the high Na/K ratio—rather than treating sodium or potassium in isolation—when developing interventions to combat CVDs in Saudi Arabia. Further comprehensive studies utilizing 24 h urinary excretion are warranted to clarify the situation across other regions of the Kingdom.

## Figures and Tables

**Table 1:** General characteristics of study participants by total sample (*n*= 600), subsample (*n* = 173), and gender.

General Characteristics ^†^	All Participants				Subsample of 24 h Urine Execration			
	Total (*n* = 600)	Females (*n* = 364)	Males (*n* = 236)	*p* Value ^†^	Total (*n* = 173)	Females (*n* = 72)	Males (*n* = 101)	*p* Value ^†^
	*n* (%)	*n* (%)	*n* (%)		*n* (%)	*n* (%)	*n* (%)	
Age (years) (mean ± SD) ^†^	23.2 (3.1)	22.9 (2.7)	23.6 (3.5)	0.004	23.8 (3.3)	23.1 (2.6)	24.3 (3.7)	0.018
Height (cm) (mean ± SD) ^†^	163.3 (8.9)	163.3 (8.9)	168.7 (9.2)	0.000	166.3 (10.4)	165.1 (9.8)	167.1(10.9)	0.193
Weight (kg) (mean ± SD) ^†^	65.7 (15.4)	62.3 (14.7)	70.8 (15.1)	0.000	69.3 (15.1)	68.1 (20.7)	70.1 (16.1)	0.484
BMI (kg/m^2^) (mean ± SD) ^†^	24.5 (4.7)	24.3 (4.9)	24.7 (4.3)	0.265	24.8 (5.3)	24.8 (6.2)	24.9 (4.6)	0.839
**BMI (kg/**$m^{2}$**)** ^‡^								
Normal weight	372 (62)	251 (68.9)	121 (51.3)	0.000	95 (54.9)	42 (58.3)	53 (52.5)	0.455
Overweight	159 (26.5)	66 (18.1)	93 (39.4)		57 (32.9)	20 (27.8)	37 (5.9)	
Obese	69 (11.5)	47 (12.9)	22 (9.3)		21 (12.1)	10 (13.9)	11 (10.9)	
**Social statues** ^‡^								
Single	510 (85)	295 (81.1)	215 (91.1)	0.002	153 (88.4)	61 (84.7)	53 (91.1)	0.416
Mired	76 (12.7)	60 (16.5)	16 (6.8)		14 (8.1)	8 (11.1)	37 (5.9)	
Divorce	14 (2.3)	9 (2.5)	5 (2.1)		6 (3.5)	3 (4.2)	11 (2.9)	
**Education** ^‡^								
Diploma	30 (5)	9 (2.5)	21 (8.9)	0.002	10 (5.8)	3 (4.2)	7 (6.9)	0.613
High school	181 (30.2)	108 (29.7)	73 (30.9)		57 (32.9)	26 (36.1)	31 (30.7)	
Bachelor degree	369 (61.5)	237 (65.1)	132 (55.9)		106 (61.3)	43 (59.7)	63 (62.4)	
Postgraduate degree	20 (3.3)	10 (2.8)	10 (4.2)		-	-	-	
**Economic statues (for family)** ^‡^								
Less than 5000 SAR	117 (19.5)	62 (17.0)	55 (23.3)	0.000	40 (23.1)	15 (20.8)	25 (24.8)	0.606
5000–10,000 SAR	157 (26.2)	106 (29.1)	51 (21.6)		47 (27.2)	23 (31.9)	24 (23.76)	
10,999–20,000 SAR	184 (30.7)	128 (35.2)	56 (23.7)		42 (24.3)	18 (25.0)	24 (23.76)	
More than 20,999 SAR	142 (23.7)	68 (18.7)	74 (31.4)		44 (25.4)	16 (22.2)	28 (27.7)	
**Smoking statues** ^‡^								
Smoker	150 (25)	49 (13.5)	101 (42.8)	0.000	57 (32.9)	12 (16.7)	45 (44.6)	0.000
Former smoker	18 (3)	9 (2.5)	9 (3.9)		9 (5.2)	2 (2.8)	7 (6.9)	
**Special diet (yes)**	100 (16.7)	59 (15.9)	42 (17.8)	0.550	21 (12.1)	6 (8.3)	15 (14.9)	0.196
**Health Statues** ^‡^								
No health issue	508 (84.7)	297 (81.6)	211 (89.4)	0.018	143 (82.7)	54 (75)	89 (88.1)	0.025
Other diseases	92 (15.3)	67 (18.4)	25 (10.6)		30 (17.3)	25.0 (12)	12 (11.9)	
**Blood pressure** ^‡^ **(*n* = 122)**								
Normal	55 (45.1)	28 (54.9)	27 (38.1)	0.159	55 (45.1)	28 (54.9)	27 (38.1)	0.159
Pre-hypertensions	56 (45.9)	20 (39.2)	36 (50.7)		56 (45.9)	20 (39.2)	36 (50.7	
Stage I	11 (9.1)	3 (5.9)	8 (11.3)		11 (9.1)	3 (5.9)	8 (11.3)	
**Physical activity** ^‡^								
Never	217 (36.2)	159 (43.4)	59 (25)	0.000	57 (32.9)	34 (47.2)	23 (22.8)	0.003
Once a week	21 (3.5)	10 (2.8)	11 (4.7)		73 (42.2)	28 (38.9)	45 (44.6)	
Twice a week	243 (40.5)	138 (37.9)	105 (44.5)		34 (19.7)	8 (11.1)	26 (25.7)	
>four times week	119 (19.8)	58 (15.9)	61 (25.8)		9 (5.2)	2 (2.8)	7 (6.9)	

^†^ Differences between gender were assessed by using independent sample t-test. ^‡^ Significant differences between the proportion of males and females in other variables were assessed using Chi2 test. SAR—Saudi Arabian Riyal.

**Table 2:** General dietary behavior across the total sample (*n* = 600), subsamples (*n* = 173), and gender.

General Dietary Behavior	All Participants			*p* Value (Mean ± 95%CI)	Subsample of 24 h Urine Execration			*p* Value **(Mean ±** **95%CI)**
	Total (*n* = 600)	Females (*n* = 364)	Males (*n* = 236)		Total (173)	Females (*n* = 72)	Males (*n* = 1 01)	
	*n* (%)	*n* (%)	*n* (%)		*n* (%)	*n* (%)	*n* (%)	
**Frequency of eating fruits per week** ^†^								
Once a week	62 (10.3)	55 (15.1)	7 (2.9)	0.000	20 (11.6)	13 (18.1)	7 (6.9)	
Twice a week	292 (48.7)	171 (46.9)	121 (51.3)		88 (50.9)	35 (48.6)	53 (52.5)	
3–4 days a week	170 (28.3)	96 (26.4)	74 (31.4)		42 (24.3)	10 (13.9)	32 (31.7)	
5–7 days a week	76 (12.7)	42 (11.5)	34 (14.4)		23 (13.3)	14 (19.4)	9 (8.9)	0.003
**Portion intake of fruit/day** *	1.4 (1.3, 1.5)	1.3 (1.2, 1.5)	1.5 (1.3, 1.7)	−0.10 (−0.35, 0.14) *p* = 0.401	1.3 (1.2)	1.1 (0.95)	1.4 (1.2)	0.32 (−0.03, 0.67) *p* = 0.073
**Frequency of eating vegetable** ^†^								
Once a week	50 (8.3)	28 (7.7)	22 (9.3)		22 (12.7)	8 (11.1)	14 (13.9)	
Twice a week	174 (29.0)	101 (27.8)	73 (30.9)		56 (32.4)	24 (33.3)	32 (31.7)	
3–4 days a week	208 (34.7)	119 (32.7)	89 (37.7)		59 (34.1)	17 (23.6)	42 (41.6)	
5–7 days a week	168 (28)	116 (31.9)	52 (22.0)	0.005	36 (20.8)	23 (31.9)	13 (12.9)	0.009
**Portion intake of vegetable/day** *	1.5 (1.4, 1.6)	1.4 (1.3, 1.5)	1.6 (1.4, 1.7)	−0.15 (−0.42, 0.13) *p* = 0.301	1.4 (1.3)	1.4 (1.3)	1.4 (1.3)	−0.06 (−0.47, 0.35) *p* = 0.773
**Frequency of fast food** ^†^								
Once a week	158 (26.3)	117 (32.1)	41 (17.4)		33 (19.1)	20 (27.8)	13 (12.9)	
Twice a week	209 (34.8)	141 (38.7)	68 (28.8)		58 (33.5)	28 (38.8)	30 (29.7)	
3–4 days a week	130 (21.7)	23 (6.3)	26 (11.1)		82 (47.4)	24 (33.3)	58 (47.4)	
5–7 days a week	97 (16.2)	65 (17.9)	32 (13.6)	0.008	-	--	-	0.004
**Eating processed food** ^†^								
Always	86 (14.3)	64 (17.6)	22 (9.3)		23 (13.3)	15 (20.8)	8 (7.9)	
Often	217 (36.2)	121 (33.2)	96 (40.7)		68 (39.3)	27 (37.5)	41 (40.6)	
Sometimes	200 (33.3)	114 (31.3)	86 (36.4)		59 (34.1)	18 (25)	41 (40.6)	
Rarely	86 (14.3)	59 (16.2)	27 (11.4)		18 (10.4)	10 (13.9)	8 (7.9)	
Never	11 (1.8)	6 (1.6)	5 (2.1)	0.014	5 (2.9)	2 (2.3)	3 (2.9)	0.044
**Number of snacks/day** ^†^								
1–2 times/day	404 (67.3)	257 (40.6)	147 (62.3)		103 (59.5)	44 (61.1)	59 (58.4)	
2–3 times/day	118 (19.7)	71 (19.5)	47 (19.9)		42 (24.3)	17 (23.6)	25 (24.8)	
3–4 times/ day	63 (10.5)	28 (7.7)	35 (14.8)		28 (16.2)	11 (15.3)	17 (16.8)	
4–5 times/day	15 (2.5)	8 (2.2)	7 (2.9)	0.033	-	-	-	0.093
**Eating snacks as a meal** ^†^								
Yes	138 (23)	87 (23.9)	51 (21.6)		51 (29.5)	23 (31.9)	28 (27.7)	
No	232 (38.7)	127 (34.9)	105 (44.5)		71 (41.0)	25 (34.7)	46 (45.5)	
Sometimes	230 (38.3)	150 (41.2)	82 (33.9)	0.050	51 (29.5)	24 (33.3)	27 (26.7)	0.354

* Differences between genders were assessed by using independent sample t-test; *p* = 0.001. ^†^ Significant differences between the proportion of males and females in other variables were assessed using Chi2 test; *p* < 0.001.

**Table 3:** Urine potassium excretion, daily potassium intake, sodium/potassium ratio, and their associations with descriptive variables (*n* = 173).

	*n*	Urine K Excretion (mmol/24 h)			Daily Potassium Intake (g)			Ratio Between Na/K		*p*-Value
		Mean (±SD)	Coef. (95% CI)	*p*-Value	Mean (±SD)	Coef. (95% CI)	*p*-Value	Mean (±SD)	Coef. (95% CI)	
**Age ^†^**	173	48.6 (23.1)	0.86 (−0.14, 1.89)	0.093	1.9 (0.89)	0.034 (−0.01, 0.1)	0.093	3.16 (1.41)	−0.10 (−0.15, 0.02)	0.149
**Gender ^†^: Male**	101	54.5 (21.3)	−13.3 (−20.1, −6.5)	0.000	2.1 (0.83)	−0.52 (−0.78, −0.25)	0.000	3.18 (1.29)	0.02 (−0.55, 0.58)	0.950
**Female**	72	40.3 (23.1)			1.6 (0.89)			3.14 (1.56)		
**BMI**	173	48.6 (23.1)	0.18 (−0.45, 0.81)	0.577	1.9 (0.89)	0.01 (−0.02, 0.03)	0.577	3.16 (1.41)	−0.02 (−0.06, 0.19)	0.314
**BMI Category ^‡^**										
Normal weight	95	4606 (21.7)	1	0.659	1.8 (0.84)		0.659	3.3 (1.45)		0.138
Overweight	57	51.9 (23.9)	3.4 (−3.9, 10.7)		2.1 (0.93)	0.13 (−0.15, 0.41)		3.2 (1.38)	−0.11 (−0.57, 0.35)	
Obese	21	48.6 (26.7)	1.8 (−8.7, 12.3)		1.8 (1.03)	0.07 (−0.34, 0.48)		2.6 (1.57)	−0.67 (−1.34, −0.01)	
**Education ^‡^**										
Diploma	10	45.6 (16.9)		0.114	1.8 (0.66)		0.114	2.4 (0.83)		0.185
High school	57	42.5 (22.8)	0.11 (−14.9, 15.2)		1.7 (0.89)	0.004 (−0.58, 0.59)		3.29 (1.48)	0.75 (−0.22,1.71)	
Bachelor degree	106	52.2 (23.1)	7.8 (−6.4, 22.1)		2.03 (0.9)	0.31 (−0.25, 0.86)		3.17 (1.39)	0.85 (−0.10, 1.76)	
**Economic statues ^‡^**										
Less than 5000 SAR	40	46.6 (20.6)		0.758	1.8 (0.80)		0.758	3.13 (1.49)		
5000–10,000 SAR	47	49.2 (30.5)	4.6 (−4.8, 13.9)		1.9 (1.19)	0.18 (−0.19, 0.54)		3.32 (1.62)	0.169 (−0.42, 0.76)	0.101
10,000–20,000 SAR	42	50.9 (20.9)	4.5 (−5.2, 14.1)		1.9 (0.81)	0.17 (−0.20, 0.55)		3.31 (1.18)	0.27 (−0.34, 0.87)	
>20,000 SAR	44	47.5 (17.9)	2.5 (−7.3, 12.3)		1.9 (0.69)	0.10 (−0.28, 0.48)		2.88 (1.29)	−0.46 (−1.07, 0.16)	
**Physical activity habits ^‡^**										
Do not workout	57	42.3 (20.2)		0.039	1.41 (0.78)		0.006	3.26 (1.42)		0.050
Workout once a week	9	58.9 (44.1)	15.2 (−0.6, 30.9)		1.68 (0.62)	0.15 (−0.27, 0.58)		3.39 (1.99)	−0.10 (−0.56, −0.44)	
Workout 2–4 times a week	73	47.2 (17.9)	3.7 (−4.1, 11.5)		2.28 (0.99)	0.66 (0.16, 1.15)		3.31 (1.32)	−0.74 (−1.35, −0.12)	
Workout daily	34	58.8 (27.3)	12.7 (2.8, 22.6)		2.69(1.00)	1.11 (0.33,1.88)		2.61 (1.32)	−0.12 (−1.12, −0.89)	
**Blood pressure ^‡^**										
Normal	55	47.1 (30.5)		0.832	1.8 (1.19)		0.832	3.2 (1.89)		
Pre-hypertensions	56	48.5 (18.3)	−2.2 (−11.2, 6.7)		1.9 (0.71)	−2.2 (−11.2, 6.7)		3.16 (1.15)	0.06 (−0.52,0.65)	
Stage 1	11	47.6 (24.3)	−3.7 (−19.1, 11.7)		1.9 (0.95)	−3.7 (−19.1, 11.7)		2.9 (1.36)	−0.17 (−1.18, 0.85)	0.895
**Total consumption of fruit and vegetables ^‡^**										
Consumed ≤ 5 portion /day	90	42.01 (16.1)	12.9 (6.6, 19.2)	0.000	1.6 (0.63)	0.50 (0.26, 0.75)	0.000	3.4 (1.7)	−0.49 (−1.04, 0.05)	0.076
Consumed > 5 portion/ day	83	55.7 (27.1)			2.2 (1.1)			2.9 (1.3)		
**Frequency of fast food ^‡^**										
Once a week	33	45.54 (19.5)			1.8 (0.76)			3.06 (1.17)		
Twice a week	58	45.99 (25.14)	−1.51 (−11.11, 8.05)		1.79 (0.98)	−0.06 (−0.43, 0.31)		3.18 (1.76)	0.13 (−0.47, 0.74)	
3- 7 days a week	82	51.67 (22.73)	1.24 (−8.04, 10.52)	0.775	0.02 (0.87)	0.05 (−0.31, 0.41)	0.775	3.19 (1.22)	0.16 (−0.45, 0.76)	0.856
**Eating processed food ^‡^**										
Always/Often	91	46.2(22.9)			1.8 (0.9)			3.3 (1.6)		
Sometimes	59	52.6 (22.4)	3.3 (−4.2, 10.7)		2.1 (0.9)	0.123 (−0.16, 0.42)		2.9 (1.1)	−028 (−0.76, 0.19)	
Rarely/Never	23	47.8 (25.2)	1.3 (−9.1, 11.6)	0.684	1.9 (0.9)	0.05 (−0.35, 0.45)	0.685	3.2 (1.4)	0.03 (−0.63, 0.68)	0.454
**Number of snacks per day ^‡^**										
1–2 times/day	103	49.1 (21.67)			1.9 (0.85)			3.02 (1.17)		
2–3 times/day	42	46.6 (23.96)	−2.53 (−10.5, 5.51)		1.8 (0.93)	−0.09 (−0.41, 0.21)		3.63 (1.84)	0.59 (0.09, 1.1)	
3–4 times/day	28	49.6 (27.09)	−1.04 (−10.4, 8.31)	0.821	1.9 (1.05)	−0.04 (−0.41, 0.32)	0.820	2.97 (1.36)	0.27 (−0.56, 0.61)	0.064
**Eating snacks as a meal ^‡^**										
Yes	51	46.8 (31.26)			1.8 (1.22)			3.3(1.77)		
No	71	50.5 (19.2)	2.6 (−5.44, 10.66)		1.9 (0.74)	0.10 (−0.21, 0.42)		3.3 (1.44)	0.04 (−0.47, 0.55)	
Sometimes	51	47.7 (18.09)	1.3 (−7.32, 9.92)	0.814	1.9 (0.71)	0.05 (−0.29, 0.39)	0.814	2.8 (1.03)	−0.43 (−0.97, 0.11)	0.145

^†^ Age- and gender-adjusted regression (model 1). ^‡^ Age- and gender-adjusted regression (model 2).

## Data Availability

The data presented in this study are available at the research center, King Abdulaziz University, or upon request from the corresponding author.
